# Raman imaging for measuring homogeneity of dry binary blend: Combining microscopy with spectroscopy for technologists

**DOI:** 10.1002/ansa.202000029

**Published:** 2020-07-11

**Authors:** Vivek Gupta, Devesh K. Pathak, Sandeep Chaudhary, Rajesh Kumar

**Affiliations:** ^1^ Discipline of Civil Engineering Indian Institute of Technology Indore Indore India; ^2^ Discipline of Physics Indian Institute of Technology Indore Indore India; ^3^ Center for Rural Development and Technology Indian Institute of Technology Indore Indore India

**Keywords:** blended mix, building construction materials, homogeneity testing, Raman mapping, Raman microscopy

## Abstract

Expanding the capabilities of Raman scattering as an analytical tool for engineering applications can optimize the technological output immensely. Understanding the homogeneity of any blended mix is one such significant parameter in the family of composite building construction materials that needs an appropriate tool for its measurement. Raman spectromicroscopy has been established here for the purpose of studying the chemical homogeneity at the microscopic scale of a dry binary blend used in the building constructions as an example. In this study, two waste stone powdered materials, obtained from western Indian stone fields, have been characterized in their respective unmixed forms using Raman spectroscopy up to an extent so that the same can be developed as a microscopic tool to clearly “see” the chemical homogeneity of a mixture. A step‐by‐step study has been carried out by first, simply making a physically separated and identifiable boundary of the two materials followed by obtaining a Raman line image. The Raman line map could clearly identify the boundary, which otherwise was not possible to appreciate visibly. The same recipe has been extended to study the homogeneity of a binary mixture (blended in 1:1 ratio), using a Raman area map. The novelty of the work lies in the advancement in the analytical tool's family to see the chemical homogeneity of building construction materials at the microscopic level. Chemical imaging using Raman spectroscopy has been demonstrated as a simple tool to understand the homogeneity of the dry binary blend, which was not possible by other simple techniques. Using Raman area mapping proves to be a quick, valuable, and effective tool for measuring the homogeneity of the blended mixes at the microscopic scale and important for application in building construction materials.

## 1 INTRODUCTION

Any experimental technique is chosen for a specific purpose based on its ability to deliver the desired information with accuracy. The best among all techniques has a tendency to keep evolving so that its applicability can be expanded as per the expansion of other fields. It becomes more important for technological applications where due to advancements therein, the characterization and diagnosis are needed, which are dynamic processes. Characterization tools that can cope up the above challenge can be said to be a versatile one. Raman spectroscopy, developed based on a light scattering^1^, ^2^ phenomenon, is one such technique that has potential applications in the areas across the fields spreading from physics, chemistry, and engineering.^3^, ^4^, ^5^, ^6^ Developing an analytical tool based on such scientific technique, to resolve the engineering challenges, will be a significant contribution to both the fields of analytical sciences and engineering.

In construction engineering, measuring the homogeneity of construction materials is a key industrial challenge for quality control engineers.^7^ The homogeneity can be measured by confirming the presence of its constituent elements uniformly throughout the matrix at any discussed scale. The homogeneity, in such materials, can be considered at two different scales: (a) macroscopic scale and (b) microscopic scale. Additionally, the homogeneity measuring tools can be classified in terms of physical homogeneity and chemical homogeneity based on the property of the material used to identify its constituent elements in the matrix. Physical homogeneity is measured based on the distinct physical characteristics of constituents such as particle size, optical reflection, color, and other hardened properties of a resulting matrix such as density and pore distribution, whereas the chemical homogeneity is measured based on the distinct chemical characteristics of constituents such as elemental composition and vibrational characteristics of bonds. At the macroscopic scale, usually physical homogeneity is examined. In construction materials, uniform distribution of aggregates throughout the matrix is considered as a measure of physical homogeneity,^8^ which is considered essential to achieve optimum mechanical and durability properties in the cementitious mixes.^9^, ^10^ However, in some cases, where the ingredients are having similar physical characteristics, measuring the macroscopic homogeneity is not so easy.

The physical homogeneity, at the macroscopic scale, is often evaluated through the optical image analysis,^11^ electric resistivity measurements,^9^ rheological performances,^12^ and sometimes, can be quantified as the degree of segregation in an easy manner. Researchers have also used some high‐end methods based on the gamma‐ray attenuation^13^ and the ultrasonic pulse velocity^14^ tester to determine the homogeneity of cementitious mixes, which only indicate the physical homogeneity at a macroscopic level based on the variation in density and compaction of the material. Besides this, López and Sarli^15^ used a different methodology to determine the homogeneity of colored mortars based on CIELAB (color space defined by the International Commission on Illumination) and the color difference formulae. However, CIELAB has certain limitations when it comes to a blended mix having similar‐colored materials. In another study, Liu et al^16^ used an imaging method based on X‐ray computed tomography and fractal theory to assess the homogeneity of asphalt concrete. Even these methods (CIELAB and tomography) require high‐end scientific knowledge and sophisticated setup and still has access only to the physical homogeneity at the macroscopic level. There is a scarcity of simple tools measuring the chemical homogeneity at the microscopic level in the literature.

At the microscopic level, scanning electron microscope along with energy‐dispersive X‐ray spectroscopy (EDS) is generally used to identify the presence of constituents and the chemical composition based on the morphology of particles and the obtained EDS spectra, respectively.^17^, ^18^ These tools can measure the homogeneity of the complex hydrated phases (calcium hydroxide [CH] and calcium silicate hydrate [CSH] phases) present in the materials. However, it requires certain sample preparation and measurements need to be carried out in vacuum conditions. Practically, in porous samples, it takes hours to achieve the required vacuum conditions. Therefore, an easy technique is required that can test the chemical homogeneity at the microscopic level in blended building materials to establish the optimized mixing of different compositions, and thus help to ensure the quality of the blended recipe.

On the other hand, the binder phase in the hardened cementitious matrix not only contains the cementitious binding material (cement or lime) but may also contain the silt^19^, ^20^ and other solid additives^21^ (eg, gypsum) with similar particle size. As an example, in the cement industry, cement is dry blended with fly ash, stone wastes, and other pozzolanic materials to reduce the overall carbon footprint of cement.^22^, ^23^, ^24^ Similarly, in paint industries, stone waste generated from dimensional stone processing industries is used as filler based on the mineralogical characteristics. Recently, Gupta et al^25^, ^26^ reported the importance of blended binders and stone wastes as construction material and characterized them using Raman spectroscopy. Such stone wastes may have similar morphology and particle size distribution therein, and it may be difficult to evaluate their homogenous mixing in the presence of pigments through the existing physical imaging technique. The stone waste can be used in a blended mix in a more efficient way if the homogeneity can be ensured chemically.

Raman spectroscopy is a powerful technique for identifying the chemical bonds^27^ and their localization in the mix. Therefore, Raman chemical mapping can be a better solution to handle this challenge as materials may be distinguished by spectroscopy based on their mineralogical or chemical characteristics, and the same can be mapped for being used as readily available in image form for technologists or engineers. This technique is being extensively explored in the field of pharmaceutics and food industry for quality control‐related applications in the manufacturing of medicinal tablets and instant food mixes.^28^, ^29^, ^30^ This technique has been evaluated as a process analytical tool to determine the end point of the mixing for pharmaceutical blends.^31^ Riolo et al^31^ compared the Raman spectroscopic data with the data obtained by conventional high‐performance liquid chromatography (HPLC) method based on the standard deviation analysis and found that the Raman data are more substantial than HPLC ones. Besides this, the analysis of homogeneity based on the high‐end technical spectra is difficult to understand by the semi‐ or nontechnical user involved in the construction and allied building product manufacturing industries. Alternatively, the colored images are easy to understand.

In the present study, Raman imaging has been established as a simpler and reliable tool to check the chemical homogeneity of the dry blended mix at a microscopic scale, first time for building construction materials. First, it has been established that the constituents can be chemically differentiated based on their Raman spectra. Second, the Raman area maps are developed, and chemical homogeneity has been measured by confirming the presence of constituent elements at the microscopic level in the developed Raman area maps. The novelty of the work lies in the advancement in analytical tools family to see the chemical homogeneity of building construction materials at the microscopic level. The proposed Raman area mapping based on chemical characteristics of ingredients can represent true homogeneity of the mix more accurately up to microlevel and is easy to understand even for nontechnical people involved in the construction and allied building product manufacturing industries.

## 2 EXPERIMENTAL METHODS

### 2.1 Materials

Two different waste stone powders were collected from dimensional stone processing units in western Indian stone fields, considering the importance of stone wastes as supplementary cementitious materials. The collected stone powders were named as S‐I and S‐II throughout the present manuscript, where S‐I stone powder belongs to the waste generated during the processing of “Dholpur stone,” whereas S‐II stone powder belongs to the waste generated during the processing of “Jaisalmer stone.” The names have been assigned based on the names of stone's origin places “Dholpur” and “Jaisalmer” in the western state of “Rajasthan” in India. Both the stone powders deferred in their color; S‐I was pinkish, whereas S‐II was yellowish in color.

### 2.2 Measurement mode

**FIGURE 1:**
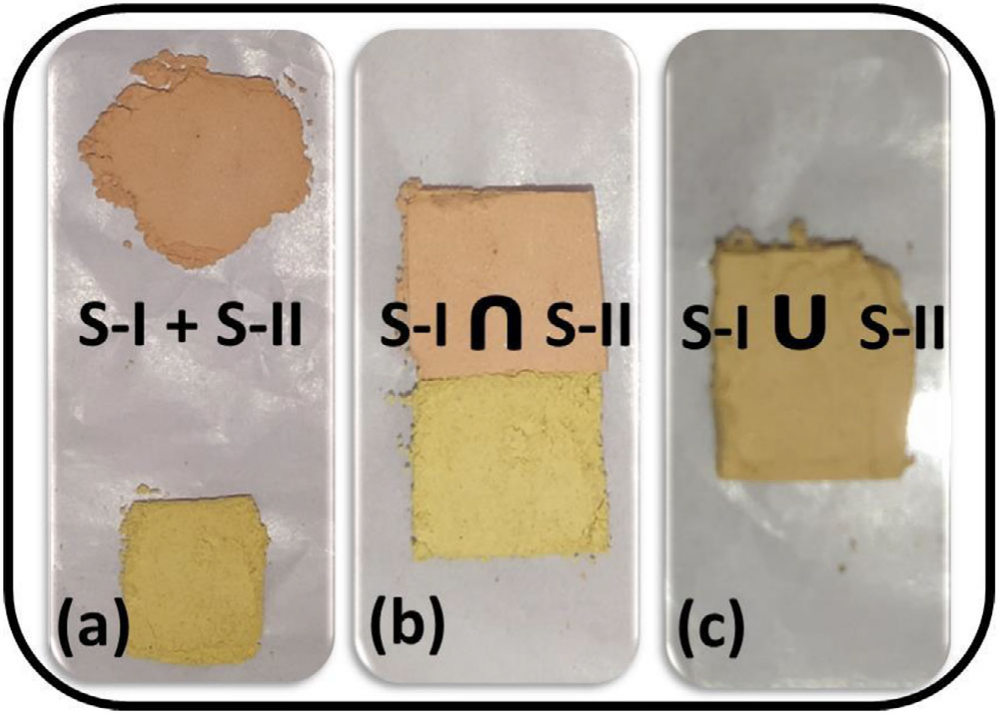
Step by step methodology for establishing the tool for measuring the chemical homogeneity. Actual images of stone powders in (A) separate heaps, (B) separated by a physical boundary, and (C) in blended/mixed form

The Raman characterization of powdered samples in dry form was carried out using “Labram HR” (JY‐ Horiba make) Raman spectrometer in backscattering geometry using a 633‐nm excitation laser source with 50× objective. The experiment was planned in the following three stages. In the first stage, the characteristic peaks of individual material were identified using the independent spectra of each material. In the second stage, the materials were brought together and allowed to form a common material boundary. In the third stage, both the materials were blended gently, and Raman area mapping was done in the selected area. The various arrangements of stone powders are as shown in Figure 1. All the Raman spectra and images have been recorded using a laser power of approximately 2 mW using an appropriate neutral density filter to avoid heating of the samples. The integration time was fixed at 10 s (two accumulations) for taking each Raman spectrum. The observed Raman spectrum has been rectified from the cosmic shower. The Raman images have been captured by recording multiple Raman spectra from a set of points on a line (area) by scanning the Raman laser on the sample with a step size of 2 μm. These spectra are then stitched in the form of an image by representing the Raman intensity as a proxy color by the Raman software.^32^, ^33^ The Raman images have been taken by recording Raman spectra from 64 points in the selected area. It took a maximum of 25 min to record the high‐resolution Raman image.

### 2.3 Methodology

Samples were oven‐dried at 50°C till constant weight was achieved and prescreened from 300‐μm sieve. For characterization in separate heaps (Figure 1A), both powders were directly taken on glass slides separately, and their individual spectra were collected. For characterization in junction arrangement (Figure 1B), a separator was used, and solid powder samples were arranged at different sides of the separator. After placing the powdered samples, the separator was removed and pressed gently against each other with another glass slide for leveling and better resolution of the spectra. Spectra were taken across the junction made on a line passing through both the materials at multiple points. For characterization in blended form, both solid powders were gently but thoroughly mixed together for sufficient time, and a pinch of powder was pressed in the same manner as above (Figure 1C). The spectra of the blended mix were collected at multiple points in a selected area. Such spectra were taken at five randomly selected regions, and two‐dimensional chemical Raman spectral mapping was obtained to verify the chemical homogeneity through confirming the presence of both the individuals in the blended mix.

## 3 RESULTS AND DISCUSSION

### 3.1 Raman characterization of stone powders in separate heaps

**FIGURE 2:**
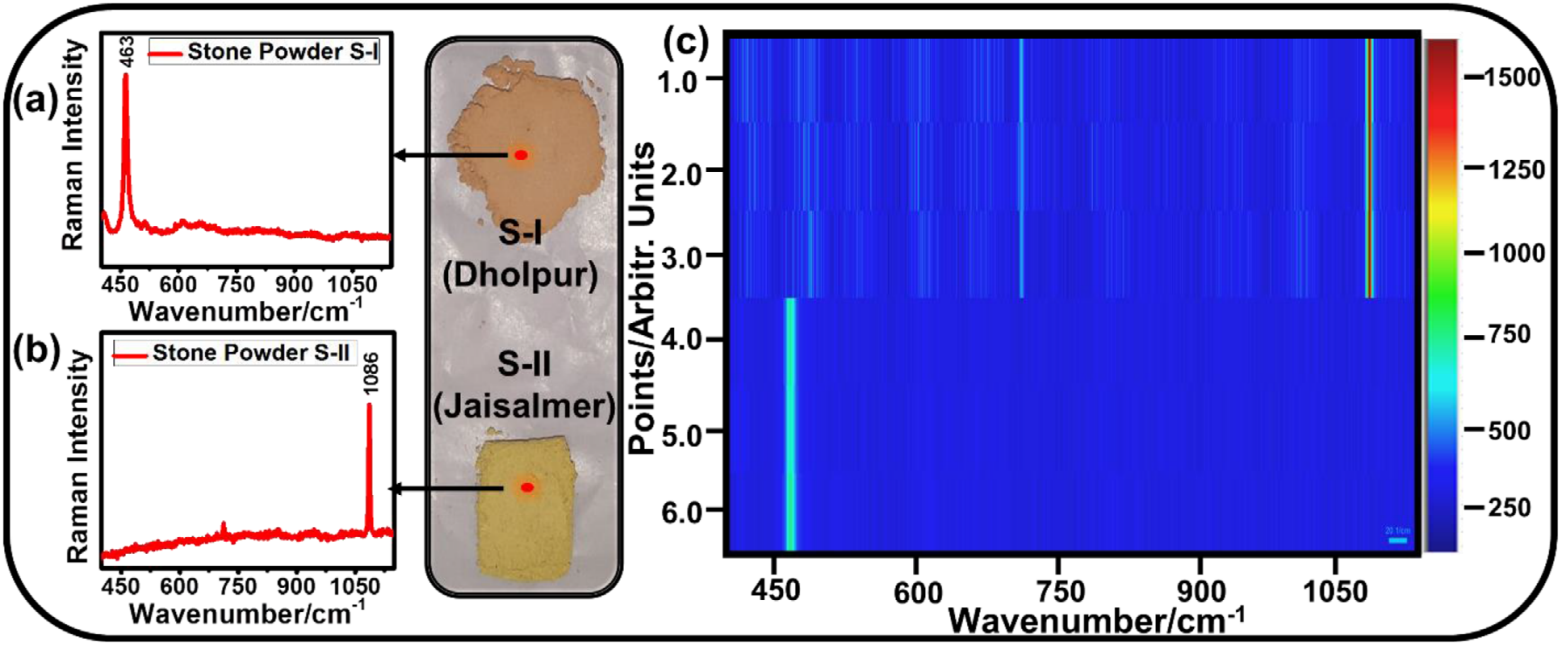
Raman spectra of the two stone powders in separate heaps (A) S‐I (Dholpur) and (B) S‐II (Jaisalmer) and (C) corresponding Raman point map at multiple selected points on separate heaps

The two raw waste powder samples (S‐I and S‐II) have been characterized using Raman spectra that show distinct spectral features (Figures 2A and 2B). In stone powder S‐I, a clear and sharp peak at 463 cm^−1^ is observed, representing the presence of quartz mineral phase,^34^ whereas in stone powder S‐II, a sharp peak at 1086 cm^−1^ has been observed, which can be attributed to calcite mineral phase.^35^ In cementitious mixes, CSH is the major reaction product that contributes mainly to the mechanical and durability properties to the hardened product.^36^, ^37^ The raw materials to produce CSH may be supplied through two different source materials, namely, Ca‐ source and Si‐ source. Hence, the selected stone powders predominantly resemble the field of building construction materials. With a broader goal to image these two materials, a Raman point map (Figure 2C) has been recorded by collecting Raman spectra (Figures 2A and 2B) from six different points (three each from S‐I and S‐II).

The obtained map (Figure 2C) can be correlated with the constituents’ representative Raman spectra, which is represented as a line pixel on the map. A correlation between the spectra and map clearly shows the presence of calcite‐rich mineral from point 1‐3, whereas the presence of quartz‐rich mineral from point 4‐6. It means that a mapping truly represents the spectroscopic information related to the chemical composition and makes it simple for analysis. Characterization done in the separate heap has been found important to define the representative characteristic peak for the further stage of experiments.

### 3.2 Raman characterization of stone powders separated by a physical boundary

**FIGURE 3:**
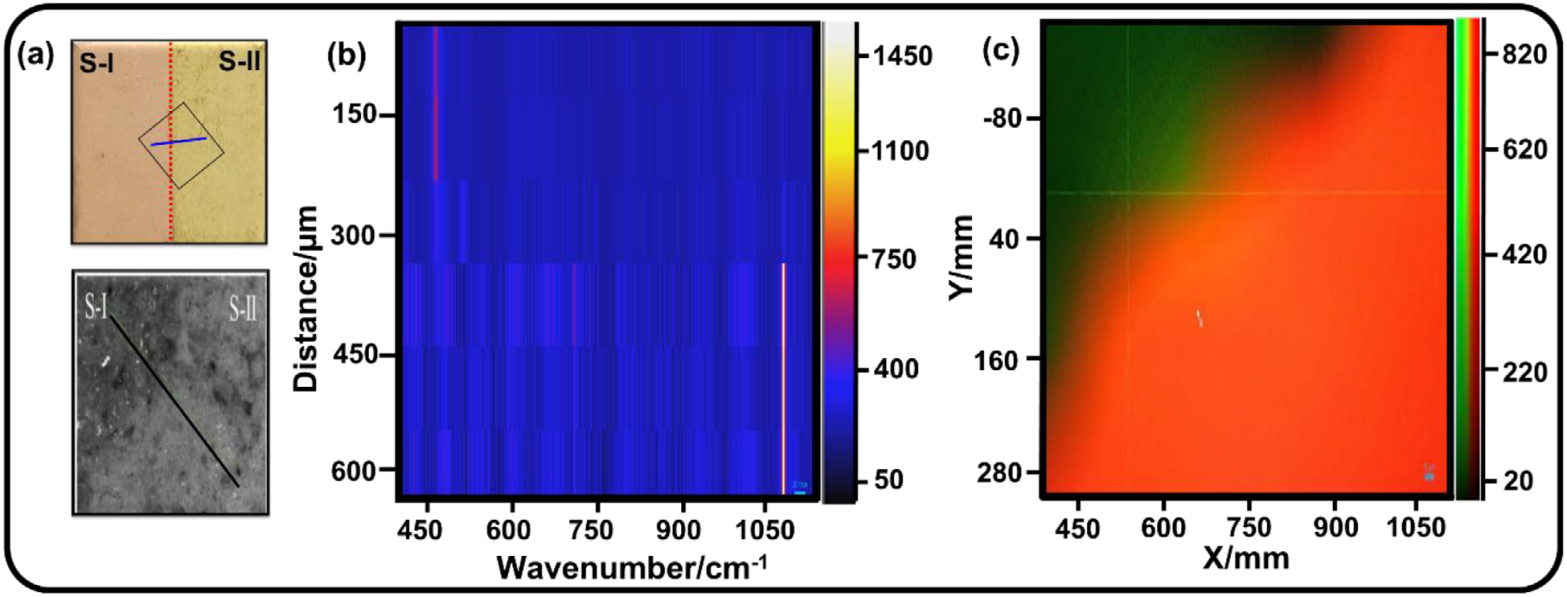
(A) Actual photograph and corresponding microscope image along with Raman (B) line scan (C) area scan for solid powders S‐I and S‐II kept by forming a boundary

Proceeding a step further toward the development of the Raman mapping tool, Raman map of the sample through Raman scanning on a line as well as an area crossing the sample boundary (Figure 1B) has been recorded. The scheme along with actual images (through naked eye and microscope) of the sample, on which Raman measurements have been carried out, is shown in Figure 3A. The Raman line map (Figure 3B) resembles the one obtained from the case when it was collected from samples separated as heaps (Figure 2C) with the only difference in the *y*‐axis where the line scan shows the distance scanned by the Raman probe. Here again, the two materials show a very clear boundary in the Raman map near *y* = 325 μm (Figure 3B), showing the region separating the two materials, silica rich (S‐I) and calcite rich (S‐II). A line scan of two different powders separated by a thin boundary clearly establishes that Raman spectra of selected materials collected at different sides of the boundary did not show any mutual interference indicating that there was no effect of the material present on the other side of the junction. Further, to confirm the same once again, Raman spectra were obtained by scanning the Raman laser probe on an area across the boundary, as shown in the scheme (Figure 3A). The corresponding Raman area image (Figure 3C) shows the Raman micrograph with a clear boundary separating the two samples (S‐I and S‐II). The green portion on the Raman image highlights the presence of silica‐rich material (S‐I) as identified by a 463 cm^−1^ peak. On the other hand, the red portion on the Raman image highlights the presence of calcite‐rich material (S‐II) as identified by a 1086 cm^−1^ peak. The area scan reestablishes the fact that there was no mutual interference of selected materials separated by thin junction during the collection of Raman spectra. It may be noted that Raman mapping of the selected area, developed in the present study, is easy to understand even for nontechnical persons involved in the manufacturing industry to identify the two different materials separated by a thin boundary. It is worth mentioning here that the materials’ arrangements, as discussed in the above two situations, do not belong to the actual scene where it is actually mixed in the desired ratio to get the necessary strength of the material. The applicability of the above mentioned Raman mapping tool will be studied below on the actual blended mix of two chemically different materials at the microlevel that are of technological importance.

Hence, in the next stage, a binary blend comprising S‐I and S‐II in equal quantities has been prepared and was then subjected to the Raman scan.

### 3.3 Raman characterization of stone powders in blended form

**FIGURE 4:**
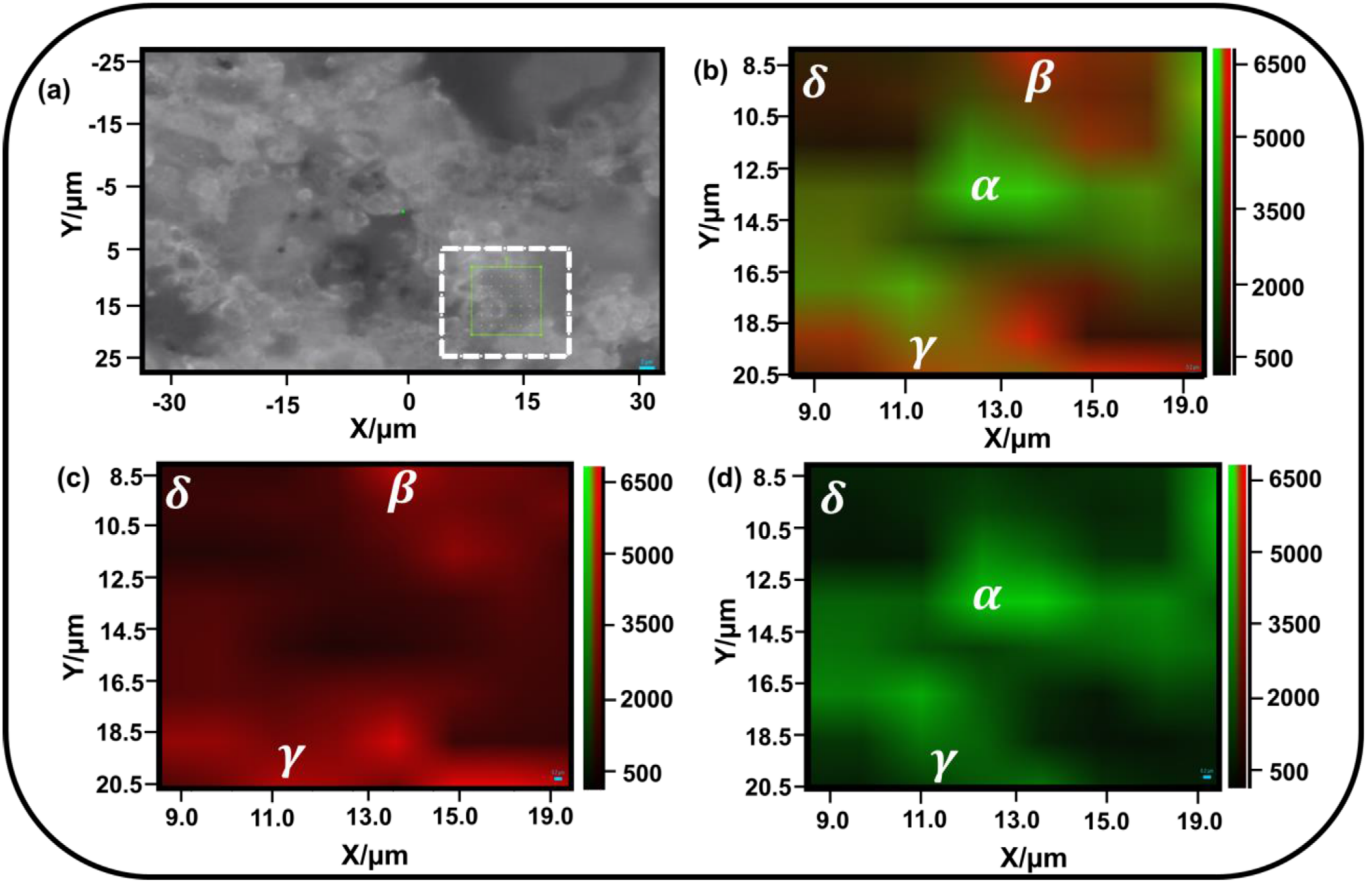
Raman micrograph from the blend made by two different solid powders S‐I and S‐II from selected portion shown on optical image (A), with (B) complete Raman image, (C) filtered Raman image highlighting silica‐rich region, and (D) filtered Raman image highlighting calcite‐rich region

Finally, Raman image from the blended mix, the final ready‐to‐use form, was captured from the materials obtained after mixing the two (Figure 1C) from an area shown in the optical photograph (Figure 4A), as seen under the Raman microscope. Five different areas were selected randomly on different samples for analysis and validation purposes. The mix is expected to have both the materials (S‐I and S‐II) present, and if the same is reflected in the Raman microscopic image, it will be advantageous as the optical images (Figures 1C and 4A) are unable to distinguish the nature of the mix. Raman micrograph from one location (selection in Figure 4A) confirms the presence of both the materials at the microlevel (Figure 4B). Randomly mixed red and green regions on the Raman image (Figure 4B) correspond to S‐I and S‐II rich areas, respectively, which are randomly mixed. It is important here to mention that the same could not be distinguished through an optical microscope (Figure 4A) or the naked eye. Furthermore, to identify the presence of individual samples (S‐I or S‐II) in the selected region, Raman image has been filtered to show individual constituent as can be identified through the two unicolor Raman images (Figures 4C and 4D). The filtered unicolor Raman image of the blended mix for S‐I (Figure 4C) and S‐II (Figure 4D) clearly confirms the presence of both the materials in the selected area. To further elaborate, four different areas are clearly marked in Figure 4B as *α*, *β*, *γ*, and *δ*. The area α denotes the calcite rich region, whereas the area β denotes the silica rich region. The presence of both the elements at one location results in yellowish color, which is marked as the area γ in Figure 4B. Some dark areas can also be seen, which are marked as the area δ in Figure 4B. Such areas represent the low‐intensity region, probably due to the poor focus of laser at that point as the *z*‐focus was not used to capture the Raman image. Areas marked as *α*, *β*, *γ*, and *δ* can also be confirmed in the filtered images Figures 4C and 4D.

The presence of both the materials at all the locations represented the homogeneity of the binary blend at the microlevel. Such filtered images can be used to confirm the presence of a particular ingredient in the multi‐ingredient mix. To validate the observation, similar area maps from different regions on different samples have been taken and four such dual‐color Raman micrographs have been shown in the Supporting Information for reference. Results (Figures S1‐S4) show observations similar to the one in Figure 4, confirming the ability of Raman imaging in the homogeneity investigations.

The point, line, and area Raman micrographs from the samples mixed in three different ways could unambiguously reveal the information about the presence of different constituents and their nature of intermixing. Raman image could distinguish the different constituents that were otherwise not possible to be seen separately using the optical micrograph or the naked eye. This three‐stage experimentation helped to develop Raman mapping as a chemical characteristic‐based homogeneity measurement tool, which can represent the true homogeneity even at the micro level, and the separate materials can actually be “seen.” In building construction materials, dry blending is an important operation performed during the production of blended cements,^38^ pre‐mixed mortars,^39^ various solid chemical grouts,^40^ composites for waterproofing application,^41^ and additive blending as powdered pigments.^42^ In the present study, Raman mapping successfully investigates the homogeneity of dry blended mixes, which can be useful for the abovementioned industries related to building construction materials. Because in the present study, the developed tool has been proven as an easy though the effective way to check the homogeneity in the dry solid mix, application for the hardened mortar and concrete can also be easily explored. Further, Raman spectra allow the data collection in wet materials too due to little interference caused by water. The possible data collection in the wet mix through Raman spectroscopy can be further explored for extended application in on‐line quality monitoring of various wet cementitious mixes.

## 4 CONCLUSION

A step‐by‐step analysis of Raman scattering experiments from individual constituents to a binary blended mix establishes Raman imaging, developed using this spectromicroscopic study, as a simple yet confirmative tool to confirm chemical homogeneity of technologically important construction material mixes at the microscopic scale. The three‐stage experimentation performed using two constituents, cutting waste collected from Indian stone fields, in separate heaps, in a thin boundary arrangement, and in the blended form, well demonstrated the stage‐wise efficacy of developed homogeneity tool for the dry blended mix. The three‐stage experimentation performed here exploits the chemical composition‐specific Raman characteristic peaks to identify the representative materials and accordingly “see” (in‐) homogeneity using Raman microscopy reliably. A micro‐level Raman map very clearly identifies the materials that are otherwise unidentifiable optically. The developed tool has shown the ability to represent the true homogeneity of the blended mix at the microlevel based on the chemical characteristic of the ingredient materials, which is a significant advancement in the family of analytical tools for homogeneity measurement of building construction materials. The chemical homogeneity testing tool developed here will be suitable for the wide range of applications related to quality control and on‐line process monitoring of dry blended products used in the building constructions. The developed tool has the possibility to be used for wet mixes and the hardened building construction products after conducting the required studies.

## CONFLICT OF INTEREST

The authors declare no conflict of interest.

## Supporting information

SUPPORTING INFORMATION
