# Supplementary material for: Raman imaging for measuring homogeneity of dry binary blend: Combining microscopy with spectroscopy for technologists
Source: Anal Sci Adv. 2020 Jul 11;1(2):89–96. doi: 10.1002/ansa.202000029 (PMC10989170; doi:10.1002/ansa.202000029)
Supplement: Supplementary file 1 — SUPPORTING INFORMATION [file ANSA-1-89-s001.pdf]

SUPPORTING INFORMATION

Raman Imaging for Measuring Homogeneity of Dry Binary Blend: Combining Microscopy with Spectroscopy for Technologists

Vivek Gupta[a,$], Devesh K. Pathak[b, $], Sandeep Chaudhary[a,c*], Rajesh Kumar[b,c*]

[a]*Discipline of Civil Engineering, Indian Institute of Technology Indore, Simrol, Indore 453552, India*

[b]*Discipline of Physics, Indian Institute of Technology Indore, Simrol, Indore 453552, India*

[c]*Center for Rural Development and Technology, Indian Institute of Technology Indore, Simrol, Indore 453552, India*

[$] *Authors having equal contribution*

* *email addresses: schaudhary@iiti.ac.in (SC); rajeshkumar@iiti.ac.in (RK)*

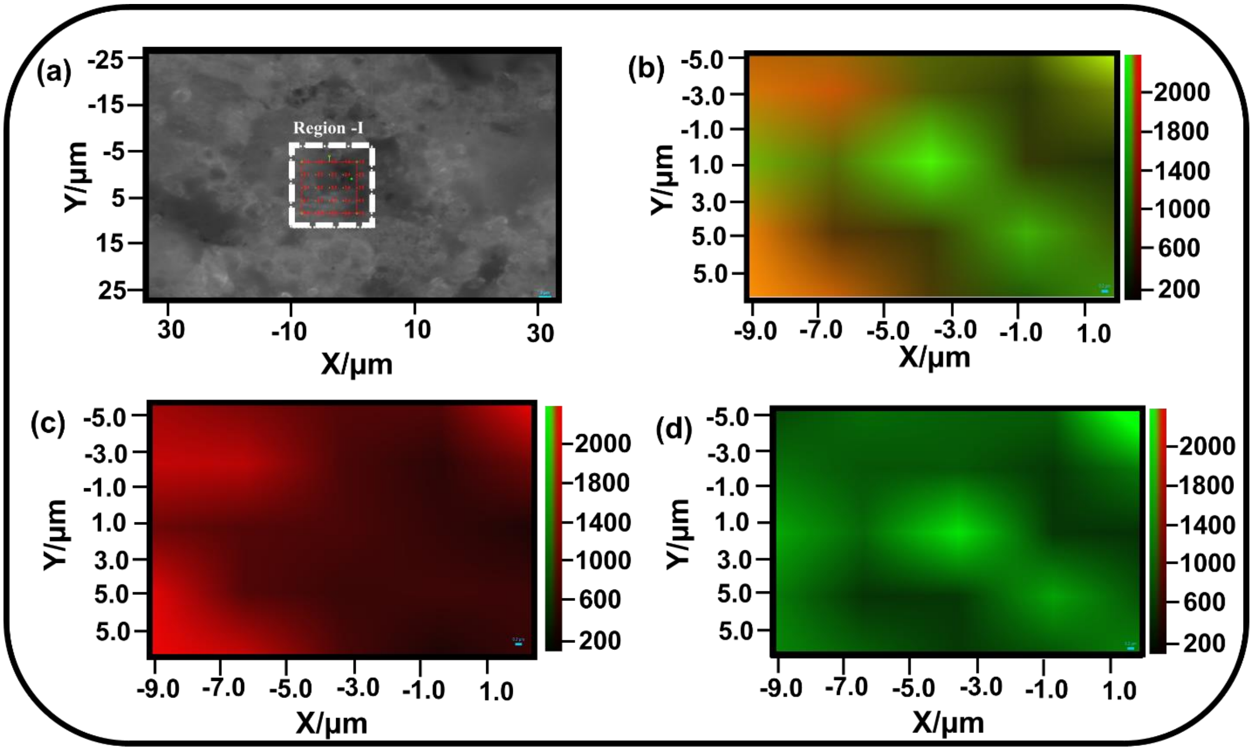

Figure S1: Raman micrograph from location-I on the blend made by two different solid powders S-I and S-II from selected portion shown on optical image (a), with (b) complete Raman image, (c) filtered Raman image highlighting silica rich region and (d) filtered Raman image highlighting calcite rich region.

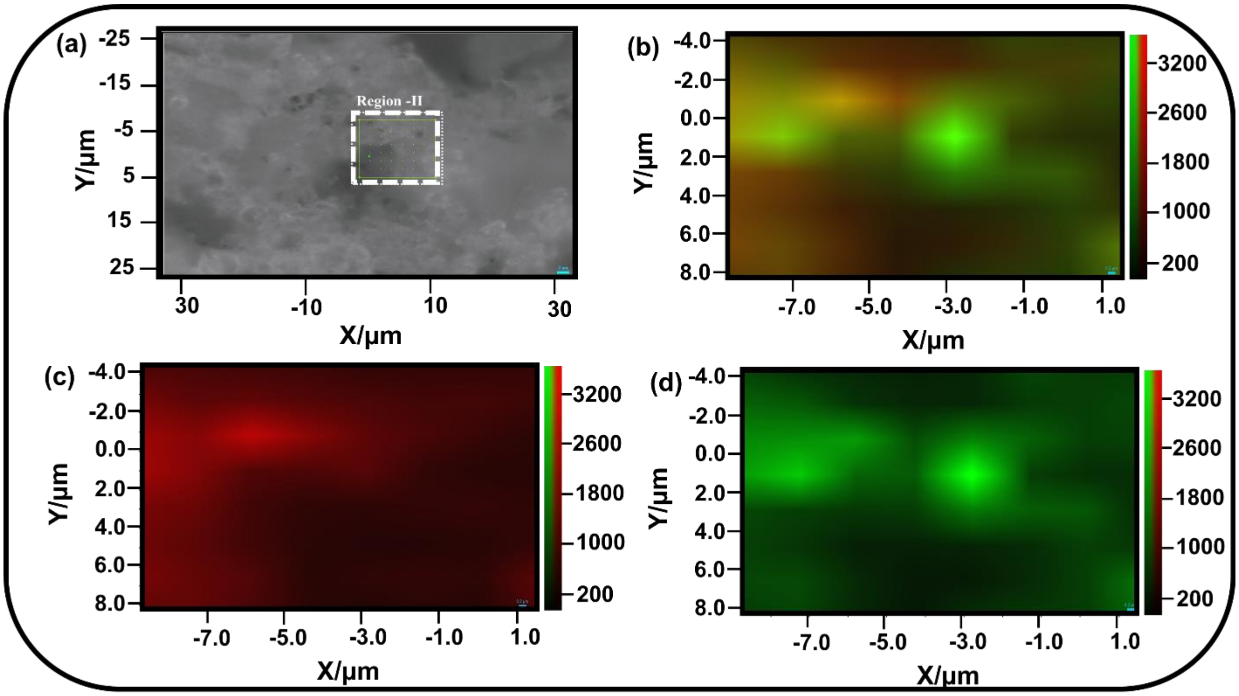

Figure S2: Raman micrograph from location-II on the blend made by two different solid powders S-I and S-II from selected portion shown on optical image (a), with (b) complete Raman image, (c) filtered Raman image highlighting silica rich region and (d) filtered Raman image highlighting calcite rich region.

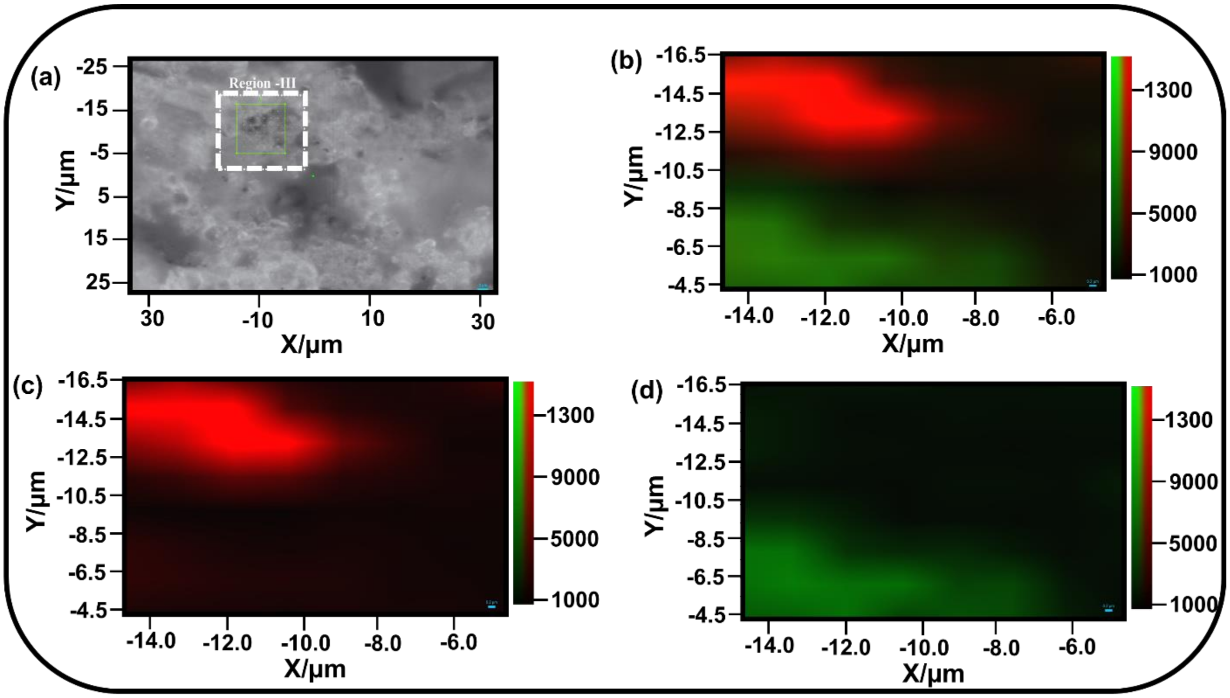

Figure S3: Raman micrograph from location-III on the blend made by two different solid powders S-I and S-II from selected portion shown on optical image (a), with (b) complete Raman image, (c) filtered Raman image highlighting silica rich region and (d) filtered Raman image highlighting calcite rich region.

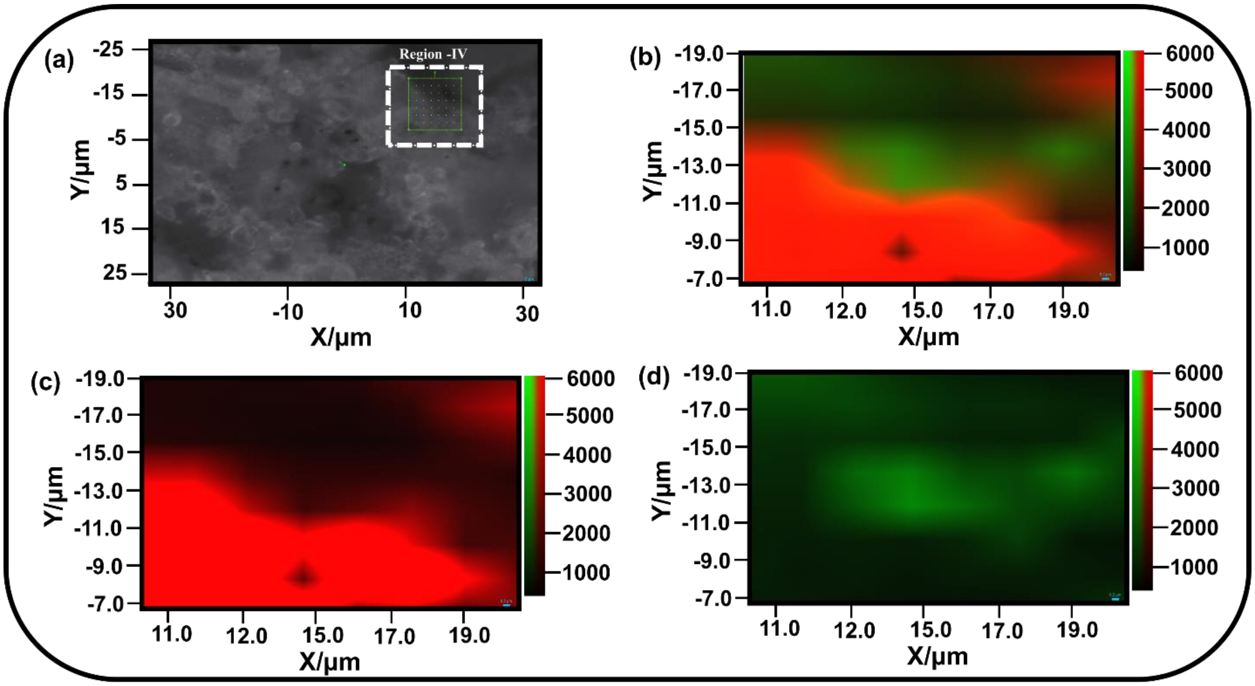

Figure S4: Raman micrograph from location-IV on the blend made by two different solid powders S-I and S-II from selected portion shown on optical image (a), with (b) complete Raman image, (c) filtered Raman image highlighting silica rich region and (d) filtered Raman image highlighting calcite rich region.
